# Characterization of Resistance to the Mexican Rice Borer (Lepidoptera: Crambidae) among Sugarcane Cultivars

**DOI:** 10.3390/insects13100890

**Published:** 2022-09-30

**Authors:** Leonardo D. Salgado, Blake E. Wilson, Hannah J. Penn, Randy T. Richard, Michael O. Way

**Affiliations:** 1 Department of Entomology, Louisiana State University Agricultural Center, 404 Life Sciences Building-LSU, Baton Rouge, LA 70803, USA; 2 Sugar Research Station, Louisiana State University Agricultural Center, 5755 LSU Ag Road, St. Gabriel, LA 70776, USA; 3 Sugarcane Research Unit, United States Department of Agriculture (USDA)-Agricultural Research Service (ARS), 5883 Usda Rd, Houma, LA 70360, USA; 4 Department of Entomology, Texas A&M AgriLife Research and Extension Center, 1509 Aggie Drive, Beaumont, TX 77713, USA

**Keywords:** *Saccharum* spp. hybrids, *Eoreuma loftini*, host plant resistance, integrated pest management

## Abstract

**Simple Summary:**

The Mexican rice borer, *Eoreuma loftini,* is one of the most important insect pests of sugarcane in the US. Management strategies that are environmentally friendly and economically profitable are needed to mitigate the pest’s impact. Cultivar resistance can become a key management strategy for Mexican rice borer control in sugarcane because it is inexpensive and easy for sugarcane growers to implement. Resistance is compatible with almost all other management strategies and minimizes further input costs incurred by the grower. However, the mechanisms of resistance are not completely understood. Elucidating which mechanisms and traits confer resistance can help sugarcane breeding programs increase the resistance level of future cultivar releases. We found that the currently grown commercial cultivars L 01-299 and HoCP 85-845 had the least amount of borer-related injury, while L 12-201 and HoCP 00-950 had the greatest level of injury. These differences appear to be related to the ability of recently hatched larvae to establish feeding rather than female preference to lay eggs on specific cultivars. Further work is needed to determine the influence of environmental factors on resistance expression and identify plant characteristics that are responsible for Mexican rice borer resistance.

**Abstract:**

Cultivar resistance is an essential management strategy for the Mexican rice borer, *Eoreuma loftini* (Dyar), in sugarcane in the USA, but resistance mechanisms are poorly understood. Resistance was evaluated among Louisiana’s (USA) commercial sugarcane cultivars and experimental clones through field screenings, greenhouse trials, and a diet incorporation assay. Cultivars L 01-299 and HoCP 85-845 had the lowest borer injury levels, while HoCP 00-950 and L 12-201 were among the most heavily injured in field and greenhouse trials. The variability of results between the two field trials suggests that a genotype × environment interaction might affect the expression of resistance. Oviposition did not differ among evaluated cultivars in the greenhouse choice study. Results from the no-choice experiment showed that neonatal establishment differed among cultivars by up to 3-fold. In a diet incorporation assay, all cultivars reduced larval weight up to 86.5% and increased days to pupation by 1.8-fold relative to the diet-only control. Collectively, these results suggest that Louisiana’s sugarcane breeding germplasm contains various resistance levels to *E. loftini,* emphasizing the importance of screening cultivars before they are released to growers. Future studies should try to determine the influence of environmental factors on resistance expression.

## 1. Introduction

In the continental United States, sugarcane is grown in Florida, Louisiana, and Texas [1]. The most important insect pests of sugarcane in Louisiana and Texas (USA) are the stem borer complex [2,3], which is formed by two species of the family Crambidae (Lepidoptera). The primary pest historically has been the sugarcane borer (*Diatraea saccharalis* (F.)), which is decreasing in importance [4].The emerging threat is the invasive Mexican rice borer, *Eoreuma loftini* (Dyar) [3,5], which is expected to increase economic losses caused by insects significantly once it has become established throughout Louisiana [5,6,7]. Since 2008, *E. loftini* has been spreading northward and eastward into Louisiana’s sugarcane and rice production regions, with infestations becoming more frequent in recent years [8,9]. More recently, *E. loftini* has become established in Florida and Georgia, where it also threatens the production of grass crops (i.e., sugarcane, corn, and sorghum) [10].

Until recently, insecticidal and biological control of *E. loftini* in Texas sugarcane has been minimal. Previous studies showed that applying insecticides to manage *E. loftini* was insufficient to increase sugar yield even with numerous applications [11,12]. Additionally, multiple attempts to import and establish parasitoids for *E. loftini* management in Texas have been unsuccessful [13]. However, modern insecticide chemistries and pheromone trap-assisted scouting have improved the chemical control of *E. loftini* in Texas sugarcane [14,15]. Nevertheless, integrated pest management (IPM) is needed to reduce reliance on insecticides and mitigate rising input costs associated with sugarcane production [7,8].

One of the most promising management tactics for *E. loftini* is cultivar resistance. Cultivars with documented resistance to *D. saccharalis* have greatly improved IPM success and reduced reliance on chemical controls methods in Louisiana sugarcane [4,5,16,17]. Cultivar resistance is particularly compatible with stem borer IPM because resistant cultivars that delay stalk entry enhance larval exposure to insecticides and natural enemies [14]. Previous studies have reported significant differences among cultivars in injury levels in the field, but resistance mechanisms are not fully understood [18,19,20,21,22].

Plant physical characteristics are important to host selection by *E. loftini* which displays a well-documented preference for oviposition in the folds of dry leaves [23,24,25]. Reay-Jones et al. (2007) [23] showed that drought-stressed sugarcane had nearly 2-fold more *E. loftini* oviposition events per plant than irrigated sugarcane. However, the influence of this behavior on preferences among modern sugarcane cultivars is unknown. Physical factors may also affect larval establishment. Traits known to affect *D. saccharalis* neonate establishment and survival, such as rind hardness [17,26] and leaf sheath tightness [27], likely also influence *E. loftini* resistance.

Similarly, nutritional content or the presence of secondary metabolites are thought to affect *E. loftini* host preferences [19]. Oviposition preference and improved larval development are associated with concentrations of key amino acids in crop and non-crop hosts [25,27]. Meagher et al. (1996) suggested that impediments to larval development in resistant sugarcane cultivars may be attributed to antinutritional compounds or other allelochemicals [19]. The influence of nutritional factors on *E. loftini* resistance among modern sugarcane cultivars, however, has not been examined.

Continuous evaluations of sugarcane cultivars and their resistance mechanisms are important for incorporating cultivar resistance into stem borer IPM programs. Resistance to *E. loftini* among modern commercial sugarcane cultivars has not been assessed and likely differs from known resistance to *D. saccharalis* based on differences in borer preferences and life histories. Therefore, the objectives of this study are to categorize resistance to *E. loftini* among modern commercial sugarcane cultivars by: (1) evaluating resistance under field conditions, (2) examining oviposition preference, (3) measuring neonate establishment, and (4) assessing larval development with a diet incorporation assay.

## 2. Materials and Methods

### 2.1. Field Screenings for Resistance

Two field trials were performed in 2016 [28] and 2020 at the Texas A&M AgriLife Research Center in Beaumont, Texas, to screen for resistance against *E. loftini* in a randomized block design replicated five times. Cultivars were randomized to 0.001-ha plots and planted on 1 Sept 2015 and 11 Sept 2019 for the 2016 and 2020 trials, respectively. Both trials included four commercial cultivars currently grown in Louisiana (HoCP 96-540, HoCP 04-838, HoCP 09-804, and L 01-299). Both trials also included HoCP 00-950 and HoCP 85-845 as *E. loftini*-susceptible and resistant standards, respectively [14,16,20,22,29]. The 2016 trial included four cultivars with limited acreage, which have not been evaluated for *E. loftini* cultivar resistance (HoCP 91-555, Ho 95-988, L 01-226, Ho 07-613), one experimental clone (HoCP 09-840), and one South African variety (N-21), which was previously shown to be *E. loftini*-resistant in Texas [22]. The 2020 trial included four additional commercial cultivars (Ho 12-615, Ho 13-739, L 01-283, and L 12-201) and two experimental clones (L 14-267 and HoCP 14-885), which have not been evaluated for resistance. Previous studies have demonstrated that high *E. loftini* pest pressure is typically present at this site [22,29,30]; thus, plots were exposed to enhanced natural infestations by suppressing predatory ant populations with insecticidal baits (Extinguish Plus^®^, Wellmark International, Schaumburg, IL, USA). The recommended sugarcane cultivation practices were followed throughout the growing season [31].

Prior to harvest on 19 Oct 2016 and 16 Nov 2020, fifteen (2016) or twelve (2020) stalks were selected randomly from each plot. Total number of internodes (2016 and 2020), bored internodes (2016 and 2020), and adult emergence holes (2016) were recorded for each sample. For the 2016 data, the relative survival of the larvae was determined as the proportion between the number of emergence holes and the number of bored internodes [20]. A relative resistance ratio, an index to incorporate bored internode and relative survival data to evaluate resistance, was used for the 2016 trial data according to the methods of Wilson et al. (2015) [22], ranking the degree of susceptibility relative to other cultivars evaluated. Data from each year were analyzed separately with generalized linear mixed models (PROC GLIMMIX, SAS Enterprise 9.4, SAS Institute, Cary, NC, USA). A binomial distribution and a logit link function were used to analyze bored internode data [32]. Cultivar was included as a fixed effect, and replication was the random effect. Denominator degrees of freedom were estimated with the Kenward-Roger method [33]. LS-Means were separated using Tukey–Kramer HSD (α = 0.05).

### 2.2. Oviposition Preference

The oviposition preference of *E. loftini* adult females was evaluated among sugarcane cultivars in a greenhouse experiment. Resistant cultivars (HoCP 85-845, L 01-299, and N-21), susceptible cultivars (HoCP 00-950 and HoCP 04-838), and commercial cultivars with unknown levels of resistance (Ho 12-615 and L 12-201) were evaluated. Three internodes per 60-L pot (n = 5/cultivar) were planted 18 Aug 2020, in a 1:2:1 combination of peat moss, autoclaved river silt, and sand. A randomized block design with five blocks was used to lay out the pots. One pot of each of the seven cultivars with a single central point of moth release was considered a block. Sixteen grams of urea (46-0-0, N-P-K) were used to fertilize the pots when the first internodes were formed. Pots were thinned out to only one stalk per pot and plant physical characteristics such as the number of green and dry leaves, stalk diameter at the third internode from the base of the plant, and plant height to top visible dewlap were recorded before insect inoculation.

*E. loftini* pupae were acquired from a colony maintained in the Entomology Department of Louisiana State University, Baton Rouge, LA, USA, following Martinez et al. (1988) [34] protocol. The colony originated with larvae collected from rice plants at the LSU AgCenter H. Rouse Caffey Rice Research Station in Crowley, LA. A multiple species artificial diet (Southland Products Inc., Lake Village, AR, USA) was used to rear insects until pupation. Forty-eight hours before release, pupae of both sexes were allowed to emerge and mate in 3.8 L plastic buckets. A random mix of thirty males and females was released on 14 Jun 2021 in each block and allowed to oviposit for four days. Oviposition events (clusters of eggs separated by more than 5 mm), eggs per event, and the total number of eggs were recorded for each plant. Data were analyzed as described before, except that a negative binomial distribution was used to analyze oviposition data with a log link function [35]. Mean separations and the denominator degrees of freedom estimation were performed as in the previous experiment.

### 2.3. Neonate Establishment

Internodes of the same seven cultivars were planted as previously described on 18 August 2020, in five 60-L pots (each pot with one cultivar was considered one repetition). Pots were thinned out to only one stalk per pot, and plant physical characteristics were recorded before the experiment started, as in the previous experiment.

On 25 January 2021, each plant was inoculated using 2.5 cm paper clips and one kraft paper roll containing *E. loftini* egg masses from the lab colony, having an average of 173.0 eggs per roll to simulate natural oviposition to the dry leaves of target internodes [14,24,36]. Neonate establishment or “boring success” was calculated as the proportion of the number of hatched inoculated eggs that resulted in larvae entering stalks as described in Salgado et al. (2022) [17].

Data were analyzed as described before using generalized linear mixed models. Cultivar was considered a fixed effect, and replication was a random effect. Neonate establishment data were analyzed with a logit link function and a binomial distribution. Gaussian distribution and an identity link function were used to analyze plant physical characteristics data. Mean separations and the denominator degrees of freedom estimation were performed as in the previous experiment.

### 2.4. Diet Incorporation Assay

Four resistant cultivars (HoCP 85-845, L 01-299, Ho 08-9003, and N-21) and two susceptible cultivars (HoCP 00-950 and HoCP 04-838) were used to evaluate the effect of leaf tissue incorporation on *E. loftini* larval development using the techniques explained in Meagher et al. (1996) and Salgado et al. (2022) [17,19]. A completely randomized design with thirty replications was used as the experimental design. Leaf-sheaths attached to the sugarcane target internode (youngest fully formed internode; [37]) were collected on 21 Jul 2020 from each cultivar from a field trial in the Sugar Research Station of the LSU AgCenter at St. Gabriel, LA. Until the start of the assay, sheaths were kept at −80 °C. Frozen leaf sheaths (n = 162 per cultivar) were freeze-dried for 72 h in a Virtis SP Scientific lyophilizer (SP Industries, Gardiner, NY, USA). Lyophilized tissue was ground for thirty seconds using default settings of the model WSG60 grinder (Waring Commercial, New Hartford, CT, USA), and a 35-mesh sieve was used to screen the resulting tissue (VWR Scientific, Seattle, WA, USA).

During artificial diet preparation, 100 mg powder/mL diet of leaf sheath tissue of each cultivar was added. To improve the blending of the diet with the sugarcane tissue, an additional 40.0 mL of deionized water was added. Thirty larvae were put in individual diet cups of insect diet with no sugarcane tissue as a control. One neonate was placed per diet cup using a fine brush, and cups were placed in an insect chamber maintained under a 14 L: 10 D photoperiod at 26 ± 1 °C, 60% ± 10% RH. Larval weight at 14 days after being starved for 3 h, days to pupation, pupal weight, and the sex of each pupa [38] were recorded.

Data were analyzed as described before using generalized linear mixed models with cultivar, as a fixed effect and replication as a random effect. The sex of each pupa and the sex × cultivar interaction were added as fixed effects for pupal weight and days to pupation models. Mean separations and the denominator degrees of freedom estimation were performed as in the previous experiment.

## 3. Results

### 3.1. Field Screenings for Resistance

**Figure 1:**
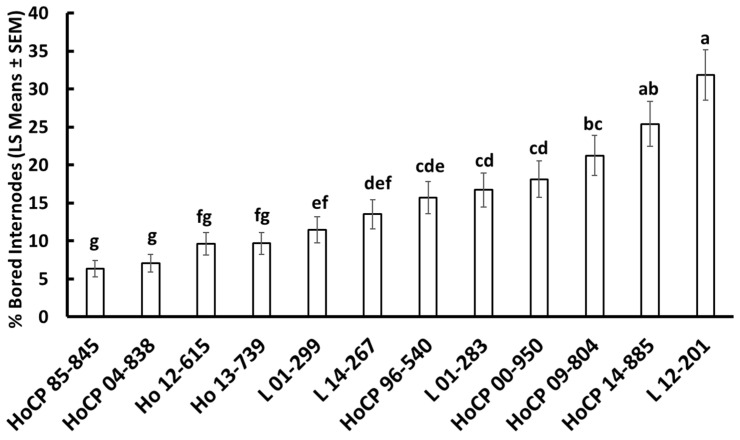
Eoreuma loftini injury among commercial and experimental sugarcane cultivars, field trial, Beaumont, Texas, 2020. Bars that share a letter are not significantly different (Tukey–Kramer HSD, α = 0.05). Standard error of means analyzed with a binomial distribution are reported.

A range of borer injury was recorded in the 2016 (Table 1) and 2020 trials (Figure 1). In 2016, cultivar influenced the percentage of bored internodes (*F* = 8.28; df = 11, 48.0; *p* < 0.001), which ranged from 0.7–5.7%. Other parameters (only measured in 2016) such as number of emergence holes per stalk (F = 0.89; df = 11, 44.0; *p* = 0.554), relative survival (F = 0.85; df = 11, 48.0; *p* = 0.595), and relative resistance ratio (F = 1.06; df = 11, 48.0; *p* = 0.415) did not differ among cultivars. HoCP 85-845, and L 01-299 received approximately 1.9-fold less injury than cultivars HoCP 09-840, HoCP 09-804, HoCP 04-838, HoCP 91-555, and HoCP 00-950. Across cultivars, approximately 5.6-fold more injury was recorded in 2020 than in 2016. Percentage of bored internodes in 2020 was influenced by cultivar (*F* = 49.84; df = 11, 48.0; *p* < 0.001) and ranged from 6.3–31.9%. Cultivars HoCP 00-950, HoCP 09-804, L 12-201, and HoCP 14-885 received approximately 3-fold more injury than HoCP 85-845 and HoCP 04-838.

### 3.2. Oviposition Preference

A total of 1,329 eggs (mean: 38.0 ± 6.5 [SE] eggs per plant) and 83 oviposition events (mean: 2.4 ± 0.5 [SE] events per plant) were recorded throughout this experiment. Number of eggs laid per plant (*F* = 0.39; df = 6, 24.0; *p* = 0.878), number of oviposition events per plant (*F* = 1.48; df = 6, 24.0; *p* = 0.228), and the number of eggs per oviposition event (*F* = 0.52; df = 6, 24.0; *p* = 0.786) did not differ among cultivars. No significant differences in plant height (*F* = 1.04; df = 6, 24.0; *p* = 0.425), internode diameter (*F* = 1.41; df = 6, 28.0; *p* = 0.246), number of green leaves (*F* = 0.76; df = 6, 24; *p* = 0.605), or number of dry leaves (*F* = 1.35; df = 6, 24.0; *p* = 0.274) among cultivars were observed.

### 3.3. Neonate Establishment

**Figure 2:**
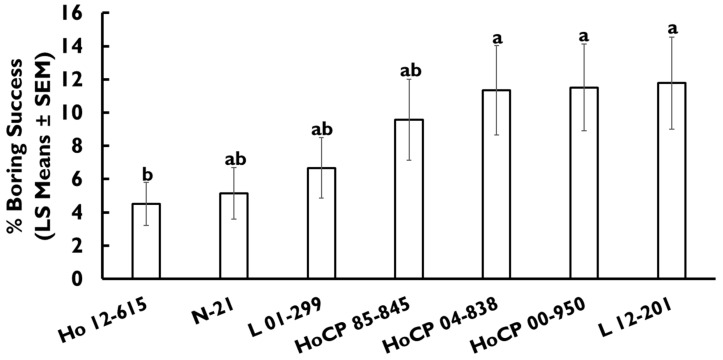
*Eoreuma loftini* neonate establishment among commercial sugarcane cultivars in greenhouse trial. Bars with the same letter do not differ significantly (Tukey–Kramer HSD, α = 0.05). Standard error of means analyzed with a binomial distribution are reported.

For this experiment, the boring success of 6000 eggs and 2408 neonates was evaluated. Cultivar had an impact on the percentage of boring success (*F* = 4.51; df = 6, 28.0; *p* = 0.003) which ranged from 4.5–11.7%. Cultivars L 12-201, HoCP 00-950, and HoCP 04-838 had 1.5-fold greater boring success relative to Ho 12-615 (Figure 2). Other cultivars had intermediate levels of injury. No differences were recorded in the percentage of larvae that hatched (*F* = 0.22; df = 6, 28.0; *p* = 0.968), plant height (*F* = 1.54; df = 6, 24.0; *p* = 0.209), internode diameter (*F* = 1.27; df = 6, 28.0; *p* = 0.304), number of green leaves (*F* = 1.20; df = 6, 24.0; *p* = 0.338), number of dry leaves (*F* = 1.03; df = 6, 24.0; *p* = 0.431), or the number of internodes (*F* = 1.35; df = 6, 28.0; *p* = 0.268) among the cultivars evaluated.

### 3.4. Diet Incorporation Assays

**Figure 3:**
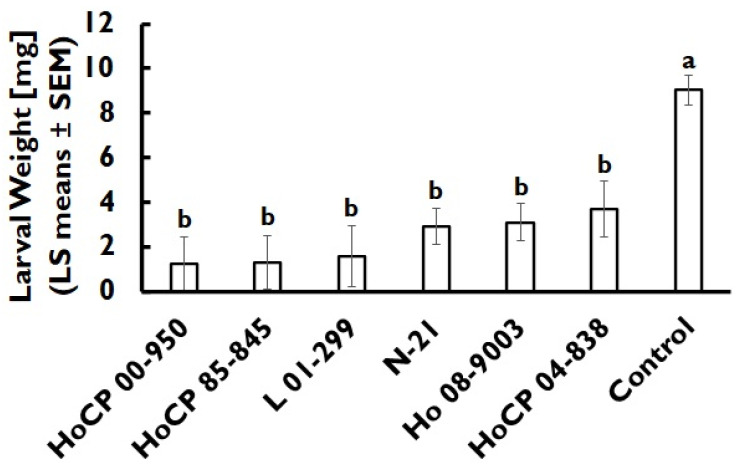
*Eoreuma loftini* larval weight as influenced by cultivar in diet incorporation assay. Bars that share a letter are not significantly different (Tukey–Kramer HSD, α = 0.05).

**Figure 4:**
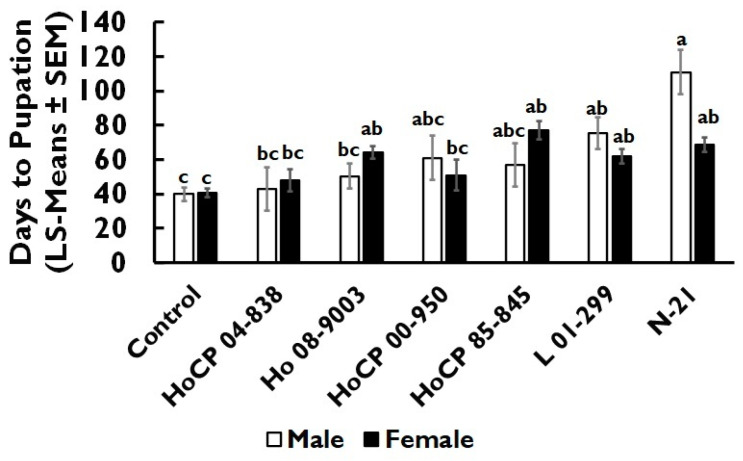
*Eoreuma loftini* days to pupation as influenced by the cultivar × sex interaction in the diet incorporation assay. Bars that share a letter are not significantly different (Tukey–Kramer HSD, α = 0.05).

All evaluated cultivars reduced larval weight by 59.2–86.5% relative to the artificial diet control but were not significantly different from each other (*F* = 12.33; df = 6, 146.5; *p* < 0.001; Figure 3). Pupal weight ranged from 29.2–39.1 mg and was influenced by sex (*F* = 21.31; df = 6, 73.2; *p* < 0.001), but was not influenced by cultivar (*F* = 0.48; df = 6, 76.0; *p* = 0.822), or the interaction of cultivar and pupae sex (*F* = 0.07; df = 6, 70.4; *p* = 0.998). Pupal weight was 2.1-fold greater in female (45.0 mg ± 2.5 mg) than in male (21.2 mg ± 4.6 mg) pupae. Days to pupation was influenced by cultivar (*F* = 13.95; df = 6, 81.0; *p* < 0.001) and the interaction of cultivar and pupae sex (*F* = 2.87; df = 6, 74.0; *p* = 0.014; Figure 4), but not by the sex main effect (*F* = 0.68; df = 6, 74.0; *p* = 0.412). For the cultivar main effect, cultivars HoCP 85-845 and N-21 had a 1.8-fold increase in days to pupation compared to the artificial diet control, while HoCP 04-838 was not significantly different from the artificial diet control.

## 4. Discussion

This work provides new insights into cultivar resistance to *E. loftini* in sugarcane and further supports the importance of factors that affect neonate establishment in stem borer resistance. Results from this study are consistent with previous work suggesting complex mechanisms of resistance to *E. loftini* exist in sugarcane cultivars [19]. This study provides new resistance evaluations for cultivars that are currently planted (HoCP 91-555, Ho 95-988, L 01-226, Ho 07-613, and HoCP 09-804), that have recently been released for commercial production (Ho 12-615, L 12-201, Ho 13-739, HoCP 14-885, and L 14-267), and one experimental clone that was not commercially released (HoCP 09-840) for the utility of cultivar resistance in *E. loftini* IPM.

Our results indicate that a range of resistance to *E. loftini* exists among modern commercial sugarcane cultivars produced in Louisiana, USA. Cultivar HoCP 85-845 can still be considered the standard for resistance in field and greenhouse studies, as seen previously [14,20,24,29]. Recently released cultivars L 12-201 and HoCP 14-885 were the most susceptible, while Ho 12-615 was among the most resistant to *E. loftini*. This suggests Louisiana’s sugarcane breeding germplasm has maintained a range of resistance levels to *E. loftini*, emphasizing the importance of screening cultivars before commercial release.

Cultivar HoCP 04-838, demonstrated to be susceptible to *E. loftini* in a 4-year study [23], was as resistant as HoCP 85-845 in our 2020 field experiment but among the most susceptible in our 2016 field experiment and no-choice greenhouse experiment. Similarly, cultivar HoCP 09-804 was moderately resistant in our 2016 experiment, but was among the most heavily injured in 2020. This discrepancy in findings between field trials suggests that a genotype × environment interaction might affect the evaluation of resistance. This is consistent with previous studies showing interactions between sugarcane cultivars and irrigation treatments influence susceptibility to *E. loftini* [24]. The influence of prior *E. loftini* injury may have also influenced resistance expression as greater levels of injury occurred in the 2020 trial. Future studies of resistance to stem borers should determine the influence of pest abundance and environmental factors in insect resistance expression.

The lack of differences in egg densities and plant physical characteristics among cultivars in the oviposition trial suggests that modern commercial cultivars may be more homogenous in traits that have been reported to influence *E. loftini* oviposition behavior [18,20,22]. However, all pots in our experiment were kept well-irrigated which may have reduced variation in dry leaves among cultivars. Because of the documented preference for dry leaves as oviposition sites, future examination of *E. loftini* oviposition preferences should include irrigated and drought-stressed treatments.

Results of the neonate establishment experiment are consistent with observations made in the field experiments and previous studies [14,37]. Cultivars HoCP 85-845, L 01-299, N-21, and Ho 12-615, had low boring success, implying that their resistance is due to low neonate establishment. The most injured cultivars in field trials, HoCP 00-950 and L 12-201, also showed higher boring success. Variation in factors not measured in this study, such as pubescence, leaf sheath tightness, and rind hardness, may have influenced neonate establishment. For instance, L 12-201 is highly pubescent on the outside of leaf sheaths [39]. While pubescence has previously been reported to deter other stem borer species’ such as *D. saccharalis* oviposition [40,41], the influence of this trait on *E. loftini* has not been examined. Still, L 12-201 is highly susceptible to *D. saccharalis* [17] and *E. loftini*, suggesting pubescence may need to occur at larval feeding sites inside leaf sheaths to confer resistance.

Larval feeding factors might play a role in conferring resistance, as evidenced by the reduction in larval weight found in the diet incorporation assay compared to the artificial diet control, as also found in other experiments [17,19]. Other studies in different insects have shown that specific amino acids in the diets were necessary for optimum growth and development [42,43,44]. Our results suggest that the artificial diet might be more nutritious than sugarcane cultivars. However, whether this difference is due to amino acid differences or feeding deterrents from the leaf sheath tissue is unclear. In contrast, in *D. saccharalis* (Lepidoptera: Crambidae), growth and development were positively impacted by the addition of 100 mg/mL of leaf sheath tissue, whereas the addition of 250 mg/mL had the opposite effect. [17]. Our study did not measure diet consumption and thus could not determine if feeding was reduced by leaf tissue inclusion. Future studies should measure the amount of diet consumed in addition to larval growth parameters to allow for differentiation between the influence of feeding deterrents and reduced nutritional value. Future studies should examine the amount of amino acids and feeding deterrents in sugarcane cultivars and their effect on *E. loftini* development.

Differences among cultivars in total larval growth and survival in our study may have been masked by the relatively greater effect of the diet-only controls. Differences were apparent, however, in relative rates of development which were consistent with differences reported among sugarcane cultivars [19] and host plant species [25] reported by previous studies. While the overall time to pupation reported here is greater than the 20–30 days reported in other studies [19,25,45], the relative differences among cultivars likely hold true. The prolonged time to pupation observed on cultivars N-21, and HoCP 85-845 suggests these cultivars may further influence field population dynamics by prolonging *E. loftini* generation time [46,47].

## 5. Conclusions

Stem borer resistance among commercial cultivars in Louisiana appears to be species-specific in some cultivars. Cultivars HoCP 85-845 and L 01-299 seem to be resistant to both *E. loftini* and *D. saccharalis* [16,48], while HoCP 04-838 appears susceptible to *E. loftini* but is consistently resistant to *D. saccharalis*. Given the increasing overlap in borer species ranges, local species composition and environmental factors should also be accounted for in consideration for cultivar screening procedures. Host-plant resistance remains an important tool in sugarcane stem borer IPM programs, but continuous resistance evaluation is needed to ensure this strategy remains successful as new cultivars are developed.

## Figures and Tables

**Table 1:** *Eoreuma loftini* injury, survival, and resistance classification among sugarcane cultivars plant cane field trial, Beaumont, TX 2016 [28].

Cultivar	Percentage of Bored Internodes (LS Means ± SEM) ^a,^*	Emergence Holes per Stalk (LS Means ± 0.10 [SE])	Relative Survival (LS Means ± 0.584 [SE])	Relative Resistance Ratio (LS Means ± 0.115 [SE])	Resistance Category ^b^
HoCP 09-840	5.7 ± 1.0 a	0.09	0.096	0.675	Susceptible
HoCP 04-838	3.5 ± 0.7 ab	0.16	0.199	0.667	Susceptible
HoCP 91-555	3.4 ± 0.7 ab	0.12	0.144	0.600	Intermediate
HoCP 00-950	3.8 ± 0.8 ab	0.28	0.222	0.600	Intermediate
HoCP 96-540	2.7 ± 0.6 bc	0.13	0.232	0.533	Intermediate
Ho 07-613	2.4 ± 0.5 bc	0.16	0.172	0.458	Intermediate
Ho 95-988	3.2 ± 0.7 ab	0.07	0.085	0.492	Intermediate
L 01-226	3.2 ± 0.7 ab	0.08	0.067	0.450	Intermediate
N-21	2.4 ± 0.6 bcd	0.03	0.073	0.433	Intermediate
L 01-299	1.0 ± 0.3 cd	0.04	0.200	0.442	Intermediate
HoCP 09-804	1.8 ± 0.4 bcd	0.08	0.096	0.358	Resistant
HoCP 85-845	0.7 ± 0.3 d	0.01	0.100	0.242	Resistant

^a^ Standard error of the mean for data analyzed with a binomial distribution. ^b^ Based on the average relative resistance ratios of the cultivars evaluated: highly resistant (0.000–0.199), resistant (0.200–0.399), intermediate (0.400–0.599), susceptible (0.600–0.799), and highly susceptible (0.800–0.999). * Means that share a letter do not differ significantly (Tukey–Kramer HSD, α = 0.05).

## Data Availability

All data are contained within the article.
